# Different effects of mating group size as male and as female on sex allocation in a simultaneous hermaphrodite

**DOI:** 10.1002/ece3.6075

**Published:** 2020-02-11

**Authors:** Masami M. Tamechika, Kohei Matsuno, Satoshi Wada, Yoichi Yusa

**Affiliations:** ^1^ Graduate School of Fisheries Sciences Hokkaido University Hakodate Japan; ^2^ Arctic Research Center Hokkaido University Sapporo Japan; ^3^ Faculty of Science Nara Women's University Nara Japan

**Keywords:** barnacle, mating group size, sex allocation, simultaneous hermaphrodite, sperm competition

## Abstract

Sex allocation theory predicts that the optimal sexual resource allocation of simultaneous hermaphrodites is affected by mating group size (MGS). Although the original concept assumes that the MGS does not differ between male and female functions, the MGS in the male function (MGSm; i.e., the number of sperm recipients the focal individual can deliver its sperm to plus one) and that in the female function (MGSf; the number of sperm donors plus one) do not always coincide and may differently affect the optimal sex allocation. Moreover, reproductive costs can be split into “variable” (e.g., sperm and eggs) and “fixed” (e.g., genitalia) costs, but these have been seldom distinguished in empirical studies. We examined the effects of MGSm and MGSf on the fixed and variable reproductive investments in the sessilian barnacle *Balanus rostratus*. The results showed that MGSm had a positive effect on sex allocation, whereas MGSf had a nearly significant negative effect. Moreover, the “fixed” cost varied with body size and both aspects of MGS. We argue that the two aspects of MGS should be distinguished for organisms with unilateral mating.

## Graphical abstract

As formalized by Charnov (1982), mating group size (MGS) is one of the most important factors affecting sex allocation of simultaneous hermaphrodites. Although the original concept assumes that the MGS does not differ between male and female functions, we found that sex allocation of a barnacle was affected differently by MGS in the male function and that in the female function.

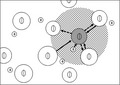

## 1 INTRODUCTION

Sex allocation in simultaneous hermaphrodites is defined as the proportion of resources allocated to the male relative to the female functions. It is an important life‐history trait affecting the fitness of an individual and hence is subject to selection pressure under given environmental conditions (Charnov, 1982). Since the first formalization by Charnov, theoretical predictions and empirical tests of the optimal sex allocation under these conditions have been a touchstone in modern evolutionary biology (Janicke et al., 2013; Leonard, 2019; Schärer, 2009; West, 2009).

**Figure 1:**
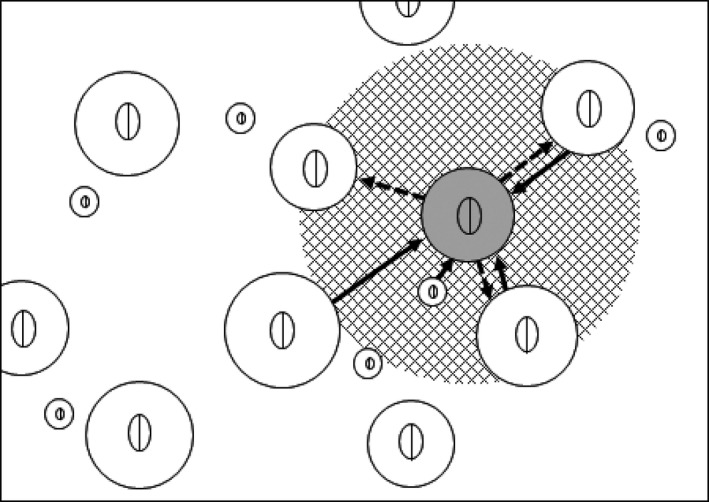
Hypothetical barnacle population consisting of large individuals (i.e., hermaphrodite phase) and small ones (i.e., protandric male phase). The area to which the penis of focal individual (gray color) can reach is shown as a meshed circle. The focal individual has different MGSs, MGSm = 4 and MGSf = 5, including itself

Irrespective of its considerable success, sex allocation theory needs refinement (Schärer, 2009). First, mating group size (MGS) has been considered an important factor that affects the optimal sex allocation. Charnov (1980, 1982) predicted theoretically that female‐biased investments are favored in small mating groups in order to alleviate local sperm competition (i.e., competition between related sperm; Schärer, 2009). Since then, empirical studies on various simultaneous hermaphrodites (e.g., Annelida, Crustacea, Platyhelminthes) have generally supported the prediction (Janicke et al., 2013; Schärer, 2009). However, Yamaguchi, Yusa, Sawada, and Takahashi (2013) pointed out that the MGS concept has two aspects, namely (a) the number of individuals to which a focal individual can donate sperm (+1 = MGS in the male function; MGSm) and (b) the number of individuals from which the individual can receive sperm (+1 = MGS in the female function; MGSf). The original MGS concept assumes that MGS does not differ between male and female functions as all individuals in the same group mate with all available individuals. Although this distinction may not be important in reciprocally mating hermaphrodites (but see Pongratz & Michiels, 2003 for an exception), these two aspects of MGS do not necessarily coincide in hermaphrodites with unilateral mating (Figure 1). This distinction is especially important in simultaneous hermaphrodites where individuals first mature as male and then become hermaphroditic (protandric simultaneous hermaphrodites; Baeza, 2007).

The second issue that needs refinement in the concept of sex allocation is the distinction between “fixed” and “variable” costs (Heath, 1977; Schärer, 2009). The fixed costs include producing and maintaining reproductive organs such as genitalia that are not consumed in each reproductive event, and the variable costs correspond to the resources allocated to produce the gametes (Heath, 1977; Schärer, 2009). These two costs will likely have different responses to environmental factors such as the two aspects of MGS; for instance, the variable cost is affected by the MGSs but not the fixed cost. Moreover, the “fixed” cost, such as the penis, varies according to the physical environments such as wave exposures in animals such as barnacles (Neufeld & Palmer, 2008). However, these are seldom distinguished in empirical studies (Lorenzi, Sella, Schleicherova, & Ramella, 2005; Schärer, 2009).

Sessilian (balanomorphan) barnacles (Cirripedia: Thoracica) are sedentary animals that deliver sperm to neighboring individuals using a long penis (Anderson, 1993; Barnes & Barnes, 1977; Murata, Imafuku, & Abe, 2001). The size of the penis is an especially important characteristic since it determines the area hence the number of individuals that an individual's sperm are delivered, hence MGSm (Neufeld & Palmer, 2008). Moreover, barnacles are the original model organisms considered in Charnov (1980, 1982) and have been used for testing sex allocation theory. They show various degrees of sex allocation, to the extent that even pure males (i.e., sex allocation = 1) and females (0) are known in several species (Darwin, 1854; Yusa, 2019; Yusa, Takemura, Sawada, & Yamaguchi, 2013). In barnacles, MGS (not distinguished into MGSm and MGSf) has different effects on sex allocation depending on the species. In the balanomorphan barnacle *Catomerus polymerus*, sex allocation is less female‐biased in large mating groups than in small groups (Raimondi & Martin, 1991) as predicted by Charnov's model. But MGS does not affect sex allocation in other species (Hoch & Levinton, 2012; Kelly & Sanford, 2010). Although the different results may reflect species difference, the degree of promiscuity and the different criteria used to distinguish small and large groups may also be relevant.

The cost of having a penis may be high in barnacles because it is several times longer than the body length in many species (Darwin, 1854; Dreyer et al., 2018; Neufeld & Palmer, 2008), and it is discarded after each breeding season at least in such species as *Semibalanus balanoides* (previously *Balanus balanoides*; Crisp & Patel, 1958, 1960; Klepal & Barnes, 1974, but not in other species; Barnes, 1992; Hoch, Schneck, & Neufeld, 2016). The penis shows phenotypic plasticity in morphology in relation to local environmental factors, such as wave strength and density (Hoch, 2008, 2009, 2010; Neufeld & Palmer, 2008). Thus, evaluating the fixed cost as male is important in barnacles. However, few studies have examined such fixed and variable costs in sex allocation (Hoch & Levinton, 2012). In this study, we examined the effects of MGSm and MGSf on the fixed and variable costs of sex allocation in a simultaneous hermaphrodite, the sessilian barnacle *Balanus rostratus*.

## 2 MATERIALS AND METHODS

### 2.1 Sample collection and rearing

*Balanus rostratus* has an annual reproductive cycle (Kado, Suzuki, Suzuki, Nanba, & Ogawa, 2009; Korn, 1985). The ovary develops most extensively from December to April (Kado et al., 2009). The development of the testis starts from February and reaches maximum before the onset of the mating season (Kado et al., 2009; Korn, 1985), which is from October to November in Northern Japan (Kado et al., 2009). Individuals keep long penises even in nonreproductive seasons (1.89–3.60 cm, mean = 2.53 cm, *N* = 36 measured in June 2018; personal observation).

**Figure A1:**
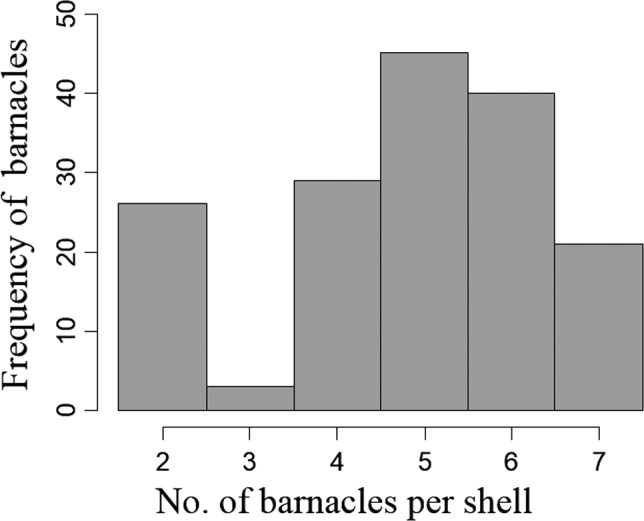
Histogram of the number of barnacle individuals per shell

Individuals of *B. rostratus* were obtained from a wave‐protected area managed by the Kawauchi Fisheries Cooperative Association (Mutsu Town, Aomori Prefecture, Japan; 41°11′50″N; 140°59′21″E) in May 2018 (*N* = 173 barnacles). These barnacles had settled naturally on the shells of the scallop *Patinopecten yessoensis* that were suspended in the sea at a depth of about 20 m in situ using hanging culture for almost 4 years. Thereafter, these shells were suspended in the sea at a depth of approximately 8 m using buoys and ropes in Hakodate City, Hokkaido Prefecture, Japan (41°56′17″N; 140°56′34″E). Shells were spaced at 20 cm intervals to avoid barnacles on different shells from interacting. Forty‐two shells, each with 2–7 barnacles (Figure A1), were reared until late September 2018 (*N* = 173 barnacles).

### 2.2 Measurements

The barnacles were frozen at the end of the rearing period, which was just before the onset of the mating season (Kado et al., 2009); therefore, the reproductive organs had completely developed but individuals had not used their gametes yet. Although they may continue to produce eggs and sperm during the mating season, it is the most suitable season to quantify sex allocation in this species.

To calculate MGS, we measured the distance between individuals (minimum distance between the two opercula) on the same bivalve shell. Then we dissected out the operculum (scutum and tergum), ovary, testis, seminal vesicles, and penis of each specimen using a nipper and tweezers. The penis was photographed with a digital camera (Stylus TG‐4, Olympus Corporation), and its length and width were measured to the nearest 10 µm using ImageJ (version 1.51j8, National Institutes of Health). Then, the operculum and ovary were each placed on an aluminum pan (35 mm in diameter, 21 mm high) that had been preheated at 480°C and preweighed with an electric microbalance (Mettler Toledo MT5) to a precision of 1 µg. The testis, seminal vesicles, and penis were each placed on a smaller aluminum pan (16 mm in diameter, 15 mm high). Then, the treated organs were dried in an oven at 60°C for 12 hr and were weighed to determine dry mass.

### 2.3 Analyses

We measured MGSm and MGSf for each individual as the number of individuals within the area reachable by its penis plus one and the number of individuals whose penises can reach the focal individual plus one, respectively. The distance reachable by the penis was considered to be 1.82 times the length of the penis of the specimen based on the elongation rate of the congener *B. glandula* in a wave‐protected shore (Neufeld & Palmer, 2008). This species has a close phylogenetic relationship to *B. rostratus* as they belong the same *Balanus balanus* group (Pitombo, 2004).

Individuals with no potential mating partner (i.e., MGS = 1) or those with some organs lost were excluded from the analyses. Following Hoch and Levinton (2012), the ovary was considered as a female variable cost. The testis and seminal vesicles were considered as a male variable cost, and the penis as a male fixed cost (Hoch & Levinton, 2012). The operculum weight was used as the index of body weight (Kado et al., 2009) because they were positively correlated (*r* = .75, *p* < .001, *N* = 162; Pearson's product‐moment correlation). Sex allocation was defined as the total male investment (testis and seminal vesicles) divided by the male and female investments (testis, seminal vesicles, and ovary). Note that the sex allocation in terms of weight as we evaluated is a relative value because we do not know the actual energetic cost invested in each sex function.

To investigate the effects of MGSm, MGSf, and that of body size, we used linear mixed‐effects models (LMMs, R package lmerTest; Kuznetsova, Brockhoff, & Christensen, 2017) in which each reproductive investment (testis + seminal vesicles, penis, and ovary) and sex allocation was treated as a response variable (*N* = 164). The MGSm and MGSf can be different as penis length varied among individuals, from 1.67 to 4.46 cm (mean ± *SD* = 2.77 ± 0.53). The effects of MGSs on all response variables were not artefacts due to multiple collinearity (generally considered to be present if Variance Inflation Factor [VIF] > 10: Chatterjee & Hadi, 2012) between MGSm and MGSf (VIF < 2.45 in all the analyses; R package car; Fox & Weisberg, 2019). The correlation coefficient *r* was 0.75, which was significant (*p* < .001; Pearson's product‐moment correlation) but was not very high as compared with the traditional assumption of *r* = 1). Additionally, the signs of estimates for MGSm and MGSf did not change between simple and multiple regressions for all response variables. All these pieces of evidence are in disfavor of the presence of multiple collinearity.

We included the shell ID (*N* = 41) as a random factor to incorporate differences in the microenvironment (e.g., number of individuals on one shell). Statistically significance was set at *p* = .05. Interaction terms were not incorporated in the models as all interactions were nonsignificant. All analyses were performed using R software version 3.6.1 (R Core Team, 2019).

## 3 RESULTS

The dry weight of the operculum ranged from 244.98 to 1,807.32 mg, and the testis and seminal vesicles ranged from 4.63 to 227.84 mg. The penis weight was much smaller, but varied greatly, from 0.33 to 2.63 mg and the length ranged from 1.67 to 4.46 cm. The ovary also varied greatly from 62.91 to 1,949.72 mg; however, all the individuals in this study had developed the ovary to some extent. Both MGSm and MGSf ranged from 2 to 7 individuals.

**Figure 2:**
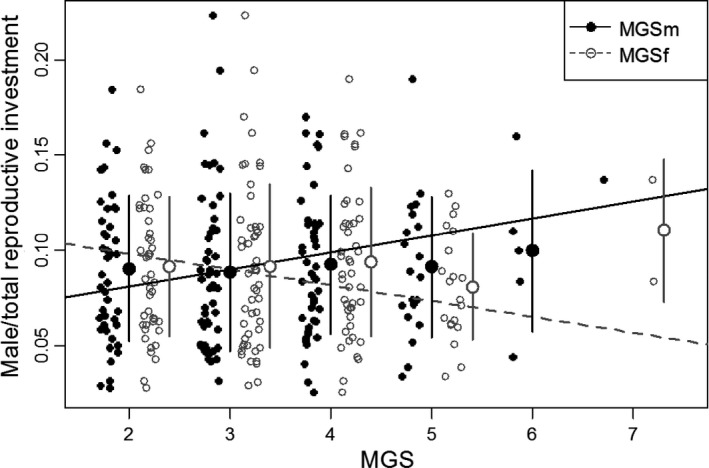
Effects of MGSm (filled circles) and MGSf (open circles) on sex allocation (male/total reproductive investment; MGSm; *p* = .0267, regression: *y* = 0.0089*x* + 0.0632, MGSf; *p* = .0501, regression: *y* = –0.0083*x* + 0.1148; LMM). Regression lines were drawn based on the mean value of body size (=1,014.18 mg operculum weight) and the values of most frequent MGS (=3, both as male and as female). Bars and large circles show standard deviation and mean value, respectively

The results of LMMs showed that both the weight of the testis and seminal vesicles (i.e., variable cost as male) and that of the penis (i.e., fixed cost as male) increased with body size and MGSm. Additionally, these male investments decreased with MGSf (Table 1). On the other hand, the ovary was only positively affected by body size and unaffected by either MGS (Table 1). Sex allocation increased with increasing MGSm and decreased almost significantly with increasing MGSf (Table 1; Figure 2). The inclusion of MGSf improved the model on sex allocation significantly as compared with the model without MGSf (Likelihood chi‐square = 3.869, *p* = .049). This is also true for model on the weight of the testis and seminal vesicles (Likelihood chi‐square = 7.546, *p* = .006) and that of the penis (Likelihood chi‐square = 10.514, *p* = .001).

**Table 1:** LMM results on effects of MGSm, MGSf, and body size on reproductive investment (testis + seminal vesicles, penis, and ovary) and sex allocation in *Balanus rostratus*

Reproductive organ	Parameters	Estimate	*SE*	*p*
Testis and seminal vesicle	Intercept	17.980	13.251	.178
	MGSm	10.799	3.629	.003
	MGSf	–10.501	3.784	.006
	Body weight (mg)	0.059	0.010	<.001
Penis	Intercept	0.275	0.104	.009
	MGSm	0.105	0.028	<.001
	MGSf	–0.097	0.029	.001
	Body weight (mg)	0.0007	0.00008	<.001
Ovary	Intercept	0.761	104.840	.994
	MGSm	40.752	30.598	.185
	MGSf	–39.898	31.512	.207
	Body weight (mg)	0.843	0.081	<.001
Sex allocation (male/total reproductive investment)	Intercept	0.1084	0.0152	<.001
	MGSm	0.0089	0.0040	.0267
	MGSf	–0.0083	0.0042	.0501
	Body weight (mg)	–0.000020	0.000012	.0930

## 4 DISCUSSION

This study shows that sex allocation responded differently to MGSm and MGSf in the barnacle *B. rostratus*. Although this needs to be interpreted with considerable care as sex allocation can be driven by only the male function, sex allocation was positively affected by MGSm, which supports the prediction of sex allocation theory (Charnov, 1980, 1982, 1987). MGSm is related to the number of eggs the focal individuals can fertilize and hence directly affects sex allocation via change in the shape of male fitness curve. Most likely, the increased sex allocation, and male investment as well, was an adaptive response to the increased number of available eggs (Charnov, 1980, 1982, 1987) with increasing MGSm. Similar increases of male allocation have been reported in many animals (e.g., Annelida; Schärer & Ladurner, 2003, Janicke et al., 2013, Chrodata; Hart et al., 2010, Platyhelminthes; Trouvé, Jourdane, Renaud, Durand, & Morand, 1999, Tan, Govedich, & Burd, 2004) including a barnacle (Raimondi & Martin, 1991).

On the other hand, when the effects of MGSm and MGSf were evaluated separately, sex allocation was affected negatively by MGSf almost significantly. Moreover, the models with both MGSm and MGSf were better than those with MGSm (and body size) alone. Combined with the fact that female investment was not affected by both MGS, the decreased sex allocation was unexpectedly owing to the decrease in the investment to male function with increasing MGSf (Table 1). Unlike MGSm which is linked to the number of available eggs, MGSf is related to the amount of sperm received. Therefore, MGSf may be a better predictor of the intensity of sperm competition than MGSm. Severer sperm competition, with the number of available eggs being constant, lowers male fitness curve and hence reducing male allocation in such cases appears to be an adaptive response. Although not known in barnacles, MGSf may also be an important factor affecting sex allocation if individuals digest received sperm for nutrition (Yamaguchi, Sawada, Nakashima, & Takahashi, 2012).

The mechanisms by which individuals sense their own MGSm and MGSf are unknown, but the barnacles may use water‐borne chemicals to collect information on the number of neighboring individuals and degrees of their ovary and testis developments. In fact, some chemicals are used to attract neighbors (Klepal, 1990) such as ascorbic acid in *Balanus* sp. (Collier, Ray, & Wilson, 1956). Barnacles even use their penises to search for functional females (Anderson, 1993; Klepal, 1990; Murata et al., 2001). Although the penis is discarded after the mating season in some congeners (Crisp & Patel, 1958, 1960; Klepal & Barnes, 1974), *B. rostratus* keep long penises even in nonmating seasons (personal observation). Thus, they are likely to collect information on mating partners and adjust sex allocation even before the onset of mating season.

The different responses of male and female outputs suggest a lack of trade‐off between male and female functions. This lack may have been caused by the absence of synchrony in the development of female and male gonads (Korn, 1985). The period of yolk formation of *B. rostratus* overlaps with the period when primary production in this area is high (i.e., winter–spring; Osaka, 1985). In contrast, the testis is fully developed in autumn, just before the onset of the mating season (Kado et al., 2009). Therefore, larger amounts of resources can be used for female investment than for male investment. Hines (1978) also indicated that egg and sperm production in some balanomorphan barnacles shows different responses to food availability. Furthermore, two other factors may be related to the lack of a trade‐off between the sexes: (a) investments in functions other than gamete production and (b) the effect of resource budget. Hermaphrodites often invest their resources to factors other than gamete production, such as parental care and mating behavior (Baeza, 2007; Lorenzi, Schleicherová, & Sella, 2006). For example, there exists a trade‐off between male behavior and egg production in the polychaete worm *Ophryotrocha diadema* (Picchi & Lorenzi, 2019; Santi, Picchi, & Lorenzi, 2018). In addition, a large variation in reproductive resource budget among individuals may mask the underlying trade‐off (Schärer, Sandner, & Michiels, 2005; Van Noordwijk & de Jong, 1986). However, we made an effort to control such effects statistically by incorporating body size and attachment site (scallop shells) in the model.

Previous empirical studies on barnacles evaluated sex allocation without considering fixed cost, or both variable and fixed costs were incorporated together as resource allocation to male function (Hoch & Levinton, 2012; Kelly & Sanford, 2010; Raimondi & Martin, 1991). This is acceptable as long as the fixed cost (a) does not vary much among individuals and (b) is negligible (Schärer, 2009). Hoch and Levinton (2012) also used the total male investment in *S. balanoides* and *B. glandula* after ensuring that the inclusion of penis weight did not change the outcome of the results, and there were no differences in the mean weight of the penis among treatments or sites. In our study, penis weight accounted for on average 1.24% (=1.00/80.75 × 100) of the total male output. Nevertheless, fixed cost varied greatly with body size and both aspects of group size. As for the fixed cost, when penis size is not negligible and there is variation in penial morphology, this investment cannot be considered as “fixed” as it varies with the environment (Schärer, 2009). The cost of building and keeping the penis can be large for barnacles living in wave‐exposed shores or in dense populations (Hoch, 2008; López, Catalán, Barriga, & López, 2014; Neufeld & Palmer, 2008). Moreover, as some barnacles mature at very small sizes (dwarf males; Yusa, 2019; Yusa et al., 2013) and the penis is renewed in each reproductive season (Crisp & Patel, 1958, 1960; Klepal & Barnes, 1974), investing in a penis is comparatively larger cost for them (Crisp, 1983; Dreyer et al., 2018). Such fixed cost may affect the optimal sex allocation, and ultimately, the evolution of sexual systems (Charnov, 1982). Therefore, it is important to correctly evaluate and incorporate fixed cost in male allocation (Michiels, Crowley, & Anthes, 2009; Schärer & Pen, 2013).

In summary, this study has shown that the effects of MGSm and MGSf on sex allocation are different, and that fixed cost is in fact highly variable. We suggest that MGSm is relevant to the number of female‐acting hermaphrodites and their eggs available to the focal individual as male, whereas MGSf is linked to the number of male‐acting neighbors and the total amount of their sperm it receives. Hence, MGSf is likely to reflect the intensity of sperm competition the focal individual experiences better than MGSm. Such distinction may also be important in other hermaphrodites with unilateral mating. Accurate evaluation of the two aspects of MGS, and fixed and variable costs, will be important in future sex allocation study.

## CONFLICT OF INTEREST

The authors declare that they have no conflict of interests.

## AUTHOR CONTRIBUTIONS

SW, YY, and MT designed the experiment; KM developed the method and MT collected and analyzed the data with help from SW; MT and YY led the writing of the manuscript. All authors contributed critically to the drafts and gave final approval for publication.

## Data Availability

All data that support this article are accessible in the Dryad repository: https://doi.org/10.5061/dryad.9zw3r229t.
